# Molecular detection reveals diverse tick-borne bacterial and protozoan pathogens in two tick species from Yingshan County of Hubei Province, China in 2021–2022

**DOI:** 10.3389/fmicb.2023.1298037

**Published:** 2023-11-23

**Authors:** Na Zhao, Kai Pan, Zhongqiu Teng, Hongliang Wang, Xue Zhang, Hongyu Ren, Lei Yi, Jia He, Kun Cai, Tian Qin

**Affiliations:** ^1^National Key Laboratory of Intelligent Tracking and Forecasting for Infectious Diseases, National Institute for Communicable Disease Control and Prevention, Chinese Center for Disease Control and Prevention, Beijing, China; ^2^Institute of Health Inspection and Testing, Hubei Provincial Center for Disease Control and Prevention, Wuhan, Hubei, China; ^3^Huanggang Municipal Center for Disease Control and Prevention, Huanggang, Hubei, China

**Keywords:** tick, *Rickettsia*, *Ehrlichi*, *Anaplasma*, *Piroplasms*

## Abstract

In this study, a total of 179 ticks infesting ruminant livestock, including 166 *Haemaphysalis longicornis* ticks and 13 *Rhipicephalus microplus* ticks were collected from Yingshan county of Hubei province, China in 2021–2022. PCR testing and sequence analysis revealed that the ticks infected with various species of pathogens including *Rickettsia* (*R. japonica*), *Anaplasma* (*A. bovis*, *A. ovis*, *A. platys*, and *Ca.* A. boleense), *Ehrlichia* (*E. minasensis* and *Ehrlichia* sp.), *Theileria* (*T. orientalis* and *T. luwenshuni*), and *Babesia* (*B. bigemina*). The infection rates of these pathogens were 0.56, 16.76, 7.26, 2.79 and 0.56%. respectively, while only 3 of 13 *R. microplus* ticks were detected to be infected wth *Ehrlichia* sp.*, A. bove*., or *T. luwenshuni*. Our results revealed that a variety of tick-borne pathogens highly carried by these ticks, specially *Ha. longicornis*. Therefore, it is necessary to make effective control of the ticks and the tick-borne diseases in the County.

## 1 Introduction

Ticks are hematophagous ectoparasites that feed on terrestrial vertebrates (Kim, 2022). They have a global distribution and are known to be the second most important arthropod vectors of human pathogens after mosquitoes (Mediannikov and Fenollar, 2014; Mansfield et al., 2017; Madison-Antenucci et al., 2020). More than 120 tick species have been found in China. *Ha. longicornis* is by far the most widely distributed and influential species in the world, exposing over 40% of the nation’s population in various counties. This has enormous implications for public health, given that *Ha. longicorni*s harbors 44 tick-borne pathogens and is a competent vector for severe fever with thrombocytopenia syndrome bunyavirus, which has been associated with a case fatality ratio of 12–50% (Li et al., 2019; El-Alfy et al., 2022). The other tick species that follow in terms of importance are *Ixodes persulcatus*, *Dermacentor nutalli*, and *Rhipicephalus microplus* (*R. microplus*) (Zhao et al., 2021). Ticks can transmit a wide variety of pathogens to both humans and animals, resulting in significant public health and economic impacts (de la Fuente et al., 2008).

Over the past 30 years, the diversity of tick-borne pathogens have been identified, including viruses, bacteria (especially *Rickettsia* and *Borrelia* spp.), protozoa, and helminths. In the mainland of China, 34 tick-borne pathogens have been identified, including eight *spotted fever group rickettsiae* (SFGR) (*R. heilongjiangiensis* (He et al., 2023), *R. japonica* (Li et al., 2019), *R. sibirica* subsp. *mongolotimonae*, *R. monacensis*, *R. raoultii*, *R. slovaca* (Tian et al., 2012), *Candidatus* Rickettsia hebeiii, and *Candidatus* Rickettsia tarasevichiae [Jia et al., 2013)], four species of genus *Ehrlichia* [*E. chaffeensis* (Cao et al., 2000), *E. canis* (Zhai et al., 2021), *Candidatus E. erythraense* (Lu et al., 2023) and *E.* sp. *Tibet* (Wen et al., 2003)], three species of genus *Anaplasma* [*A. platys*, *A. capra*, and *A. phagocytophilum* (Li et al., 2015)]; one species of genus *Neoehrlichia* (*Candidatus* N. Mikurensis). six species of genus *Borrelia*, eleven species of genus *Babesia*, and a *severe systemic thrombocytopenia syndrome virus* (SFTSV) (Tokarz and Lipkin, 2021). With social change and urbanization, humans, animals, and ticks are increasingly sharing habitats, leading to a higher likelihood of human exposure to ticks (Matos et al., 2022; Jin et al., 2023). These emerging tick-borne infections pose an increasing public health threat in China (Li et al., 2020; el-Alfy et al., 2023).

Yingshan County in Hubei Province is located within the Dabie Mountain Range. The county is mainly characterized by mountainous and forested topography, with a warm, humid climate, abundant rainfall, and distinct seasons, which are often accompanied by natural disasters. This type of climate and terrain provides suitable living and breeding conditions for ticks, increasing the risk of tick-borne diseases. Furthermore, our team previously identified a novel tick-borne pathogen in the Dabie Mountains area of China. Therefore, this study aims to investigate the epidemiology and genetic diversity of bacterial and protozoan pathogens carried by the ticks parasitizing ruminant livestock in Yingshan County, Hubei Province.

## 2 Materials and methods

### 2.1 Tick collection and identification

During 2021–2022, this study collected adult ticks in yingshan county (E 115.679, N 30.735) of Hubei Province, China. The ticks infesting ruminant livestock (cattle and goats) were collected. All ticks were morphologically identified according to previous report (Azmat et al., 2018), their species were confirmed by *COI* gene sequencing assay (Chen et al., 2010).

### 2.2 DNA extraction

All ticks were initially washed for 15 min each in a sequential manner using 5% bromogeramine solution, 75% alcohol, and PBS (Lu et al., 2023). After air-drying, they were placed in 2 mL centrifuge tubes with steel beads and 200 μL Sucrose-Phosphate-Glutamate (SPG) solution was added. Then, they were homogenized individually using a grinder (Retsch, Germany). Genomic DNA was extracted from each sample using the QIAamp DNA Mini Kit (Qiagen, Hilden, Germany) according to the manufacturer’s instructions and eluted in a final volume of 100 μL (Wen et al., 2003). All DNA samples were stored at −20°C.

### 2.3 Detection pathogens in ticks

The bacterial pathogens, including *Rickettsia* spp.*, Anaplasma* spp., *Ehrlichia* spp., *Bartonella* spp., *Borrelia* spp., and *Piroplasms*. were screened by real-time PCR (qPCR) assays with corresponding primers specific for each pathogen. A Ct cut off value of 35 was used for determining positive samples, which were subsequently subjected to semi-nested or nested PCR specific to each pathogen, the specific primers and amplified fragment sizes are presented in Supplementary Table S1.

For *Rickettsia* spp., amplification targeted the *16S rRNA* (1,200 bp), *ompA* (500 bp) *gltA* (900 bp) and g*roEL* gene (1,100 bp). For *Anaplasma* spp. and *Ehrlichia* spp., a semi-nested PCR assay targeting a 500 bp region of the 16S rRNA gene was carried out for preliminary typing, followed by genus-specific or species-specific primers targeting 16S rRNA, *gltA*, or *groEL* gene for final confirmation. To detect and characterize tick-borne protozoan pathogens, we employed a semi-nested PCR approach using a universal primer set targeting the 18S rRNA gene of *Piroplasms* (1,400 bp). The corresponding primers for amplification are listed in Supplementary Table S1.

The amplification products were verified through 1.0% agarose gel electrophoresis, and the size of DNA fragments was determined by comparing them with standard molecular size DNA ladders. PCR products with clear target DNA bands were sent to Tianyihuiyuan Biotechnology Company (Beijing, China) for sequencing.

### 2.4 Phylogenetic data analysis

SeqMan software (DNASTAR, Madison, WI) was utilized to edit and assemble DNA sequences obtained through sequencing, with a specific focus on assembling the 16S rRNA gene from *Rickettsia* spp., *Anaplasma* spp., and *Ehrlichia* spp. by merging two overlapping segments to obtain near full-length gene sequences. The resulting sequences were analyzed and compared with all available sequences in GenBank using the Basic Local Alignment Search Tool (BLAST). A neighbor-joining method was implemented in MEGA 7.0 software to construct a comprehensive phylogenetic tree, and the stability of the tree topology was evaluated by computing bootstrap support values from 1,000 replicates.

## 3 Results

### 3.1 Tick sampling and identification

**Figure 1:**
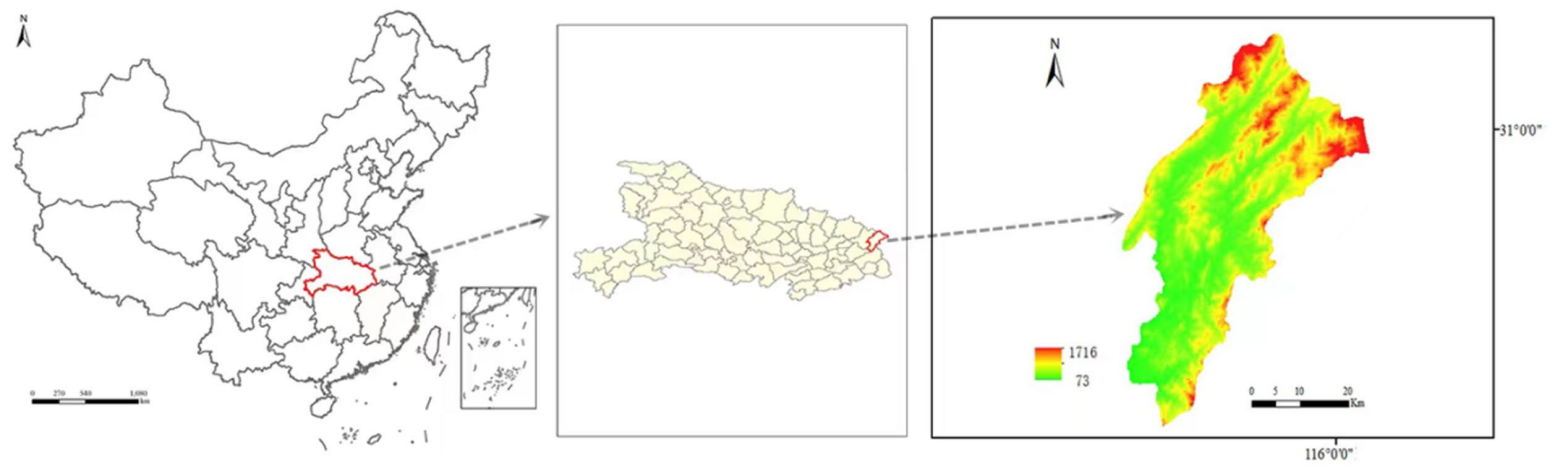
Map of the study area. Yingshan County of Hubei province, China.

**Figure 2:**
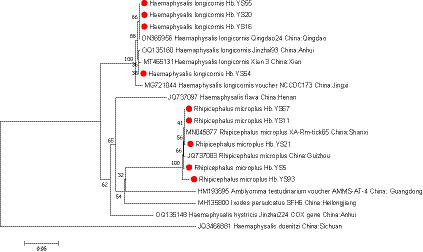
phylogenetic analysis of ticks was conducted based on the nucleotide sequences of *COI* gene. The resulting tree shows the relationship of *Ha. longicornis* and *R. microplus* with other tick species. Sequences obtained in this study are marked with a red dot before their names.

During the period of 2021–2022, a total of 179 ticks were collected from Yingshan County of Hubei province, China (Figure 1). Through meticulous morphological examination and *COI* sequence analysis, 166 ticks were identified as *Rhipicephalus microplus* (*R. microplus,* 92.73%, 166/179) and 13 ticks were identified as *Haemaphysalis longicornis* (*Ha. Longicornis,* 7.26%, 13/179). The phylogenetic tree constructed on the basis of *COI* gene sequences is shown in Figure 2; the *COI* gene sequences generated in this study were clustered with their respective homologs in two main groups corresponding to two species.

### 3.2 Pathogen diversity detected in ticks

Various bacterial pathogens, including *Rickettsia* spp.*, Anaplasma* spp.*, Ehrlichia* spp., and *Piroplasms*, were detected in ticks through PCR assays, while *Borrelia* spp. and *Bartonella* spp. was not detected (Table 1).

**Table 1:** Prevalence of tick-borne pathogens in the two ticks collected from Yingshan County of Hubei, China.

Tick species origin		Cattle	Goats		Toal (*n* = 179) /prevalence (%)
Pathogen genus species		*R.microplus* (*n* = 123)	*R.microplus* (*n* = 46)	*Ha. longicornis* (*n* = 13)	
*Rickettsia*					
	*R. japonica*	1			1 (0.56%)
*Anaplasma*					
	*A. marginale*	22	4	1	27 (15.08%)
	*A. bovis*			1	1 (0.56%)
	*A. platys*		1		1 (0.56%)
	*Ca.* A. boleense	1			1 (0.56%)
*Ehrlichia*					
	*E. minasensis*	2	1		3 (1.68%)
	*Ehrlichia* sp.	3	5	2	10 (5.58%)
*Theileria*					
	*T. orientalis*	3	1		4 (2.23%)
	*T. luwenshuni*			1	1 (0.56%)
*Babesia*					
	*B. bigemina*	1			1 (0.56%)

A total of four *R. microplus* ticks were found to carry two or three tick-borne pathogens, accounting for 2.23% of the total. Among them, two ticks (1.12%) were co-infected with *A. marginale* and *Ehrlichia* sp.; one tick (0.56%) was co-infected with *Ehrlichia* sp. and *T. orientalis*; and one tick (0.56%) carried three different pathogens, namely *A. marginale*, *Ehrlichia* sp., and *T. orientalis.*

**Figure 3:**
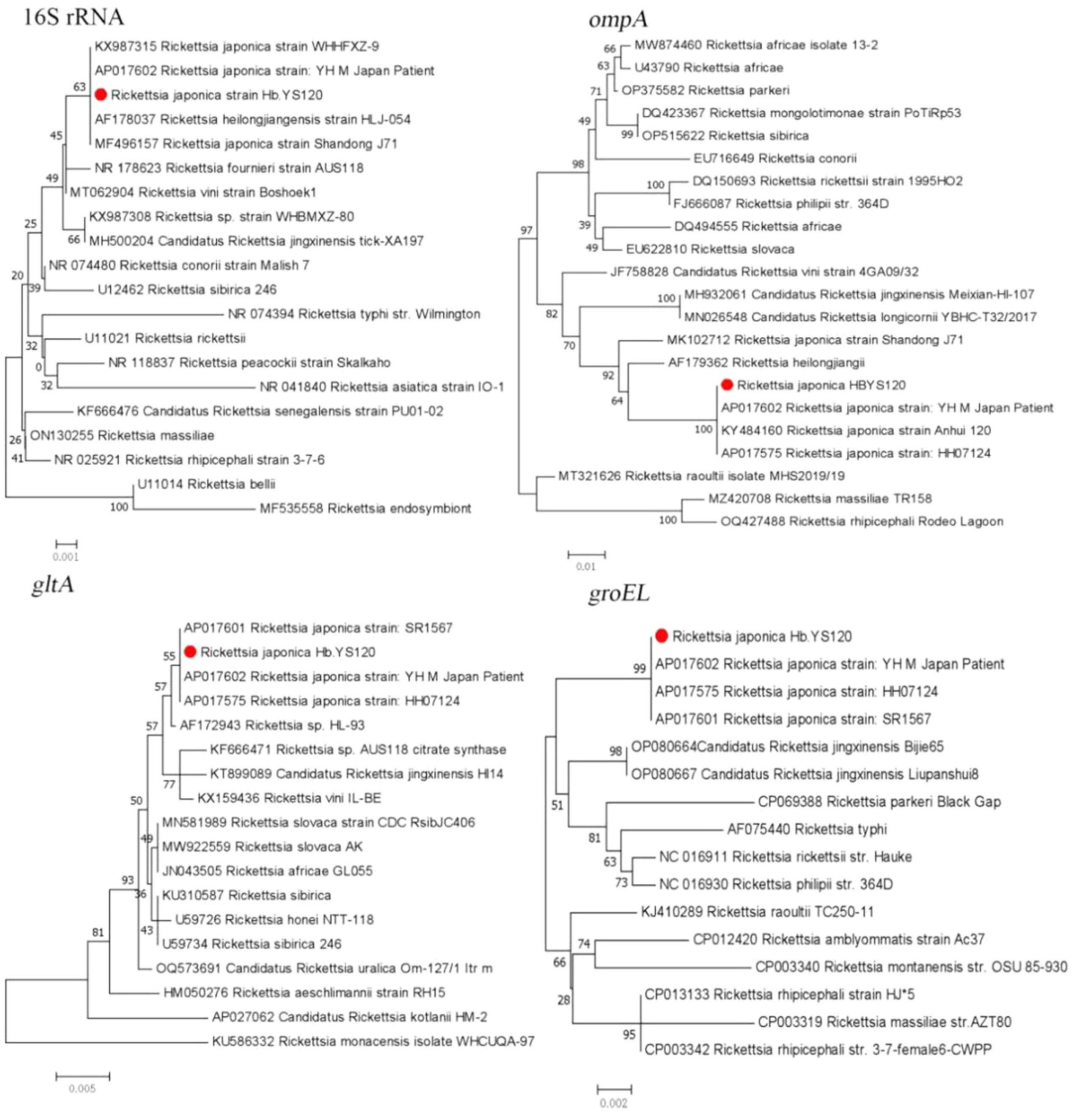
Phylogenetic trees constructed s of *Rickettsia* strains basis of the nucleotide sequences of 16S rRNA (1,200 bp), *ompA* (500 bp), *gltA* (900 bp), and *groEL* (1,100 bp) genes of *Rickettsia* strains. Sequences obtained in this study are marked with a red dot before their names.

The PCR results showed a low infection rate of *Rickettsia* spp. in the ticks, with only one *R. microplus* tick positive for *R. japonica*. The sequences of 16S rRNA, *ompA, gltA*, and g*roEL* genes obtained from the tick were 99.84 to 100% similar to those of *R. japonica* strains and 100% identical with that of *R. japonica* YHM (AP017602) (Figure 3).

**Figure 4:**
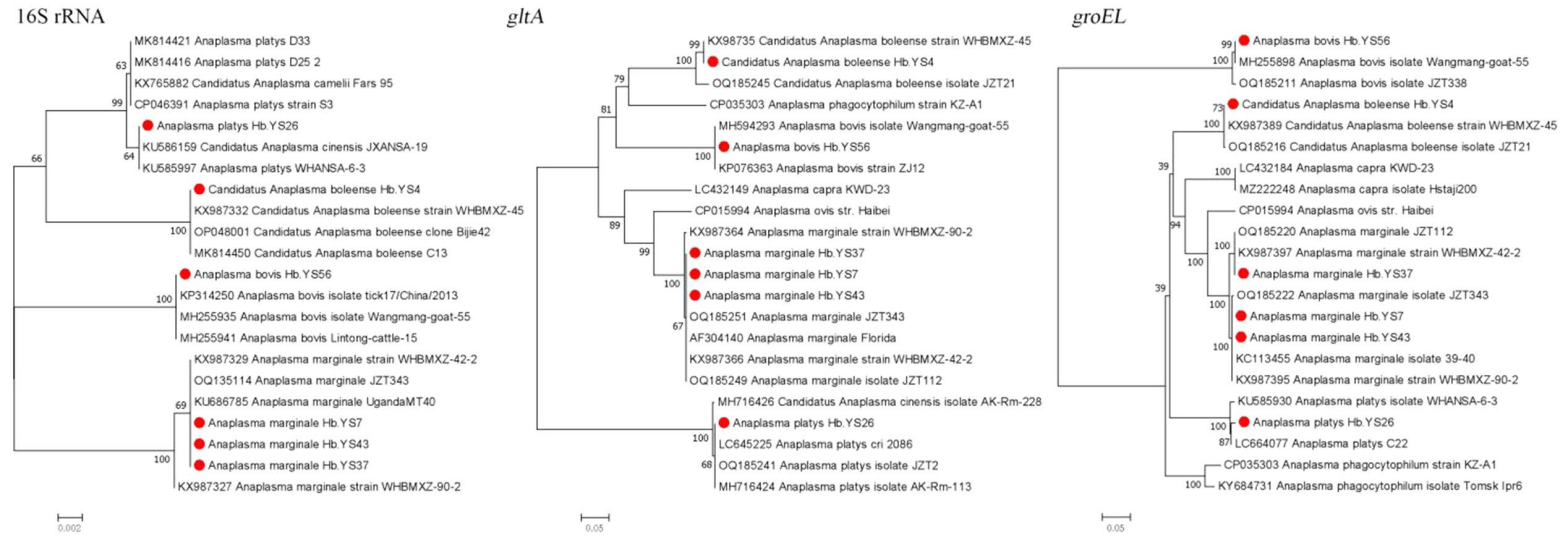
Phylogenetic trees constructed s of *Anaplasma* strains basis of the nucleotide sequences of 16S rRNA (1,400 bp), *gltA* and *groEL* genes of *Anaplasma* strains. Sequences obtained in this study are marked with a red dot before their names.

In this study, four species of the genus *Anaplasma* (*A. bovis, A. ovis, A. platys, and Ca.* A. boleense) were detected in the ticks with a positive rate of 16.76% (30/179). *A. bovis* was only detected in *Ha. longicornis*, while the other three species were detected in *R. microplus*. The genetic sequences of the 16S rRNA, *gltA*, and *groEL* genes in *A. bovis* were found to share similarities of 99.76, 100, and 100%, respectively, with those of the previously reported *A. bovis* strains, one of which was Wangmang-goat-55 strain (MH255935, MH255898, and MH594293) from china. The sequences of 16S rRNA and g*roEL* genes of *A. platys* detected in the ticks showed similarities of 99.52 and 99.78% to those (KU585997 and KU585930) from the *A. platys* strain from Wuhan City of Hubei Province, respectively. The sequences of 16S rRNA, *gltA*, and *groEL* genes of *Ca.* A. boleense exhibited similarities of 99.67, 100, and 100%, respectively, with those (KX987332, KX987358, and KX987389) of *Ca.* A. boleense strain (WHBMXZ-45) detected in *Boophilus microplus* ticks from Wuhan City of China. For the strains detected in *A. marginale*, their 16S rRNA, *gltA*, and *groEL* sequences showed the highest similarities with those (KX987327, KX987364, KX987395) of WHBMXZ-90-2 strain and those (KX987329, KX987366, KX987397) of WHBMXZ-42-2 strain from *A. marginale* in Philippines, as well as those (OQ135114, OQ135251, OQ135222) of JZT343 from *A. marginale* in China. Their similarities ranged from 99.15 to 99.98% for 16S rRNA, 99.68 to 100% for *gltA*, and 99.44 to 100% for *groEL* sequences (Figure 4).

**Figure 5:**
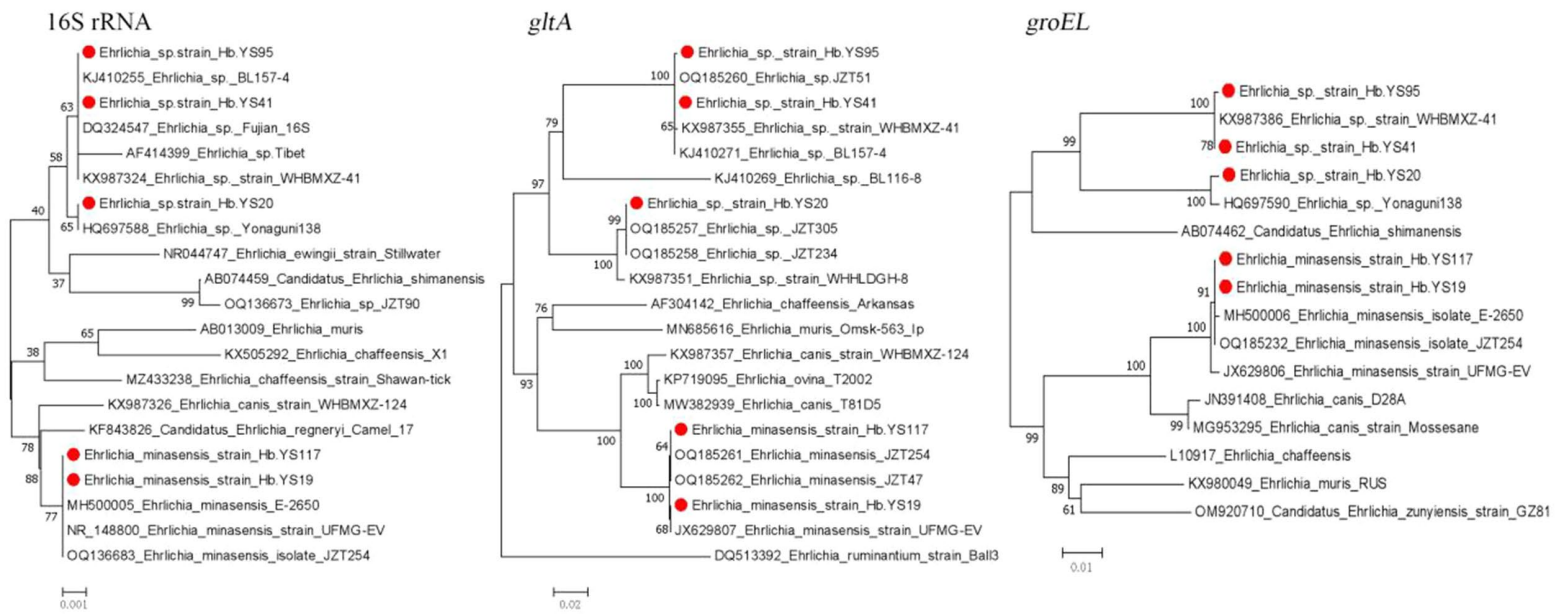
Phylogenetic trees constructed s of *Ehrlichia* strains basis of the nucleotide sequences of 16S rRNA (1,400 bp), *gltA* and *groEL* genes of *Ehrlichia* strains. Sequences obtained in this study are marked with a red dot before their names.

This study detected two species (*E. minasensis* and *Ehrlichia* sp.) of the genus *Ehrlichia*. The *16S rRNA*, *gltA* and *groEL* gene sequences of the *E. minasensis* detected in the ticks were nearly identical to those (NR148800, JX629807, and JX629806) of *E. minasensis* UFMG-EV strain isolated from *R. microplus* ticks in Brazil in 2016. Additionally, the gene sequences were highly similar to those (OQ136683, OQ185261, and OQ185232) of *E. minasensis* JZT254 strain detected in *R. microplus* ticks in China in 2021. The *Ehrlichia* sp. detected in this study were divide into two genotypes based on their 16S rRNA, *gltA* and *groEL* gene sequences in the phylogenetic analyses. One genotype shared 100% similarity in 16S rRNA and *groEL* gene with *Ehrlichia* sp. Yonaguni138 strain (HQ697588 and HQ697590) from the ticks in Japan. The other genotype showed 99.84% similarity in 16S rRNA gene with the pathogenic *Ehrlichia* sp. Tibet (AF414399). Additionally, it shared 99.56–99.70 and 99.91% similarity in *gltA* and *groEL* gene with those (KX987355 and KX987386) of *Ehrlichia* sp. WHBMXZ-41 strain isolated from *R. microplus* ticks from Wuhan city of China, respectively (Figure 5).

**Figure 6:**
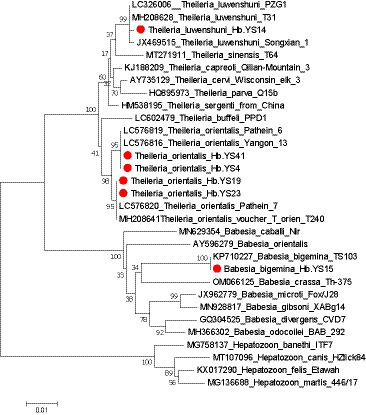
Phylogenetic analysis of the *Theileria* and *Babesia* strains based on their 18S rRNA gene sequences (1,400 bp). Sequences obtained in this study are marked with a red dot before their names.

According to Figure 6, the 18S rRNA gene sequence of *T. luwenshuni* detected in this study showed high similarity (98.77%) to that of *T. luwenshuni* T31 strain (MH208628) from *R. microplus* ticks in China and *T. luwenshuni* PZG1 strain (LC326006) from goats in Myanmar. In the phylogenetic tree, the 18S rRNA gene sequences of *T. orientalis* were classified into two genetypes, with 3–7 nucleotide differences between them. Sequencing and BLAST analysis revealed that one genotype exhibited high 18S rRNA gene sequence similarity (97.20%) to *T. orientalis* Pathein_6 strain (LC576819) from cattle in Myanmar. The other genotype showed a higher similarity (99.79%) to *T. orientalis* T240 strain (MH20864) from *R. microplus* ticks in China. Furthermore, the 18S rRNA gene sequence of *B. bigemina* detected in this study showed 99.10% gene sequence similarity to that (KP710227) of *B. bigemina* TS103 strain from the cattle in China.

## 4 Discussion

Our study utilized a molecular approach, incorporating PCR, DNA sequencing and phylogenetic analysis, to investigate the diversity of ticks and tick-borne bacterial and protozoan pathogens. During 2021 and 2022, a total of 179 ticks infesting cattle or sheep were collected from Yingshan County of Hubei Province in China, 166 of which were identified as *Ha. Longicornis* and only 13 were *R. microplus*. According to the study reports, *Ha. longicornis* is predicted to have the widest distribution in Asia, potentially impacting a population of 588 million people across 1,140 counties. And *R. microplus* holds the third position and is predicted to have the potential to affect a population of 350 million people across 678 counties in Asia (Zhao et al., 2021).

In this study, only one species of genus *Rickettsia*, *R. japonica*, was detected in a single specimen of *R. microplus*, and the sequences of 16S rRNA, *ompA*, *gltA*, and *groEL* genes detected in the tick was showed the highest nucleotide identity with those of *R. japonica* YHM strain from a patient in Japan (Akter et al., 2017). *R. japonica* is the causative agent of *Japanese spotted fever* (JSF) initially found in Japan. So far, human JSF has been found in Japan, South Korea, Philippines, Thailand, and China. From 2014 to 2021, human JSF has been reported at least hundreds of cases in mainland China, including 31 cases from Hubei province, 20 cases from the areas surrounding Jiangxi province, 20 cases from Henan province, and 18 cases from Zhejiang province in China (Li-Juan et al., 2018; Lu et al., 2018; Li et al., 2019; Zhang et al., 2023). The findings demonstrate the presence of *R. japonica* in ticks in the region, Combining our team’s recent report of the first 5 cases of JSF in Hubei Province, China, including one unfortunate death, this suggests the potentially posing health risks to the local residents (Teng et al., 2023). Therefore, it is imperative to strengthen the prevention and control measures for JSF and raise public awareness about this disease to safeguard public health.

Most of *Anaplasma* species are considered animal-specific pathogens, making them significant to public health as tick-borne bacteria. In this study, a high diversity of *Anaplasma* species was identified in the ticks collected from Yingshan County, including *A. marginale*, *Ca.* A. boleense, *A. bovis*, and *A. platys*. Among them, *A. marginale* was the most prevalent infectious tick-borned agent, infecting 15.08% of the ticks. *A. marginale* is known to be a specific pathogen of ruminants, commonly infecting cattle (Kocan et al., 2003), sheep (Yousefi et al., 2017), and goats (Barbosa et al., 2021), indicating its prevalence and potential impact on livestock in Yingshan County. As for *Ca.* A. boleense, its pathogenicity toward animals or humans is not well studied. *Ca.* A. boleense was initially identified in ticks from Bole City of Xinjiang Uygur Autonomous Region in China (Kang et al., 2014) and has been detected in mosquitoes and rodents in various provinces of China (Guo et al., 2016). Its wide host range and geographical distribution suggest that *Ca. A. boleense* merits further investigation. Furthermore, *A. platys* and *A. bovis* are considered important zoonotic pathogens. *A. platys* is widely distributed and can cause infectious cyclic thrombocytopenia in dogs and cats. The first case of *A. platys* infection in humans was confirmed by DNA sequencing in 2013 (Kang et al., 2014). *A. bovis* was traditionally considered to only infect cattle and cause bovine ehrlichiosis, which is prevalent in Africa and Asia. However, *A. bovis* was confirmed as a pathogen causing human infection in China in 2017 (Guo et al., 2016). In this study, we detected *A. platys* and *A. bovis* DNA in the ticks from ruminants, suggesting a potential risk of zoonotic transmission of *A. bovis* from livestock to farmers who are in regular contact with them.

*Ehrlichiae* are tick-borne bacteria that are responsible for life-threatening emerging human zoonoses and diseases of veterinary importance worldwide, collectively called ehrlichioses (Esemu et al., 2011). *E. minasensis* has a wide distribution and has been reported in multiple locations around the world, including Canada, Brazil, Pakistan, Malaysia, China, Ethiopia, and the Mediterranean island of Corsica. These findings indirectly suggest that *E. minasensis* may be transmitted by multiple tick species (Moura de Aguiar et al., 2019). In this study, we detected DNAs of *E. minasensi*s in three *R. microplus* ticks in Yingshan County, of Hubei Province, China. which expands the distribution of *E. minasensis* in China and indicates it may transmission among local cattle populations. Further investigations of *E. minasensis* are warranted to assess the current situation in the region. This study also detected two uncultured Ehrlichia species, one of which is *Ehrlichia* sp. Yonaguni138 that was initially found in ticks from Japan (Matsumoto et al., 2011). The other species clusters together with *Ehrlichia* sp. Tibet, a tick-borne pathogen detected in ticks or animal hosts in the phylogenetic tree (Wen et al., 2002).

Piroplasms, including *Theileria* and *Babesia*, are apicomplexan parasites and may also be transmitted by ticks, and they are found worldwide infecting both wild and domestic animals. Among these, *T. luwenshuni* stands out as a highly pathogenic pathogen specifically affecting goats and sheep, leading to significant economic losses for farmers and livestock industries. *T. orientalis* is the economically predominant pathogen for bovine theileriosis in China, but it usually causes mild or asymptomatic disease (Rizk et al., 2015; Wang et al., 2020). In the context of bovine babesiosis in China, *B. bigemina* is considered one of the major causative agents. Infections with *B. bigemina* are characterized by a low level of parasitemia, which may contribute to its persistence infection and pose challenges in diagnosis and control (Niu et al., 2015; He et al., 2021). The impact of piroplasms on livestock production is particularly pronounced in developing countries. These parasites cause considerable morbidity and mortality in affected animals, resulting in reduced yields of meat, milk, and other livestock by-products. *Theileria* and *Babesia* infections pose significant challenges to the sustainable growth of the livestock industry, as they not only lead to economic losses but also hinder the overall well-being of animal populations.

## 5 Conclusion

In summary, we have identified numerous bacterial and protozoan pathogens in *R. microplus* and *Ha. longicornis* ticks collected from free-ranging ruminant livestock in Yingshan County of Hubei Province in China. Some of these bacteria have been reported to infect humans. This finding has raised concerns as there is close spatial proximity and contact between humans and tick hosts in Hubei Province, indicating a potential risk of human exposure to these tick-borne pathogens in the region. This is of significant importance for public health and disease prevention. Our research provides valuable references and data support for relevant authorities to develop effective prevention and control measures, and to strengthen surveillance and early warning systems for tick-borne diseases.

## Data availability statement

The original contributions presented in the study are publicly available. This data can be found at: https://www.ncbi.nlm.nih.gov/; OR467404-OR467409, OR501452-OR501460, OR508717-OR508728, OR555720-OR555744.

## Author contributions

NZ: Writing – original draft. KP: Resources, Writing – review & editing. ZT: Methodology, Writing – original draft. HW: Resources, Writing – review & editing. XZ: Data curation, Methodology, Writing – review & editing. HR: Data curation, Writing – review & editing. LY: Resources, Writing – review & editing. JH: Methodology, Writing – review & editing. KC: Resources, Writing – review & editing. TQ: Conceptualization, Writing – review & editing.

## References

[ref1] Akter A. Ooka T. Gotoh Y. Yamamoto S. Fujita H. Terasoma F.. (2017). Extremely low genomic diversity of *Rickettsia japonica* distributed in Japan. Genome Biol. Evol. 9, 124–133. doi: 10.1093/gbe/evw304, PMID: 28057731 PMC5381555

[ref2] Azmat M. Ijaz M. Farooqi S. H. Ghaffar A. Ali A. Masud A.. (2018). Molecular epidemiology, associated risk factors, and phylogenetic analysis of anaplasmosis in camel. Microb. Pathog. 123, 377–384. doi: 10.1016/j.micpath.2018.07.034, PMID: 30053605

[ref3] Barbosa I. C. André M. R. Amaral R. B. D. Valente J. D. M. Vasconcelos P. C. Oliveira C. J. B.. (2021). *Anaplasma marginale* in goats from a multispecies grazing system in northeastern Brazil. Ticks Tick Borne Dis. 12:101592. doi: 10.1016/j.ttbdis.2020.101592, PMID: 33099171

[ref4] Cao W. C. Gao Y. M. Zhang P. H. (2000). Identification *Ehrlichia chaffeensis* by nested PCR in ticks from southern China. J. Clin. Microbiol. 38, 2778–2780. doi: 10.1128/JCM.38.7.2778-2780.2000, PMID: 10878087 PMC87030

[ref5] Chen Z. Yang X. Bu F. Yang X. Yang X. Liu J. (2010). Ticks (acari: ixodoidea: argasidae, ixodidae) of China. Exp. Appl. Acarol. 51, 393–404. doi: 10.1007/s10493-010-9335-2, PMID: 20101443

[ref6] de la Fuente J. Estrada-Pena A. Venzal J. M. Kocan K. M. Sonenshine D. E. (2008). Overview: ticks as vectors of pathogens that cause disease in humans and animals. Front. Biosci. 13, 6938–6946. doi: 10.2741/3200 18508706

[ref7] El-Alfy E. S. Abbas I. Baghdadi H. B. El-Sayed S. A. E. Ji S. Rizk M. A. (2022). Molecular epidemiology and species diversity of tick-borne pathogens of animals in Egypt: a systematic review and meta-analysis. Pathogens 11:912. doi: 10.3390/pathogens11080912, PMID: 36015033 PMC9416077

[ref8] el-Alfy E. S. Abbas I. Elseadawy R. Saleh S. Elmishmishy B. el-Sayed S. A. E. S.. (2023). Global prevalence and species diversity of tick-borne pathogens in buffaloes worldwide: a systematic review and meta-analysis. Parasit. Vectors 16:115. 2023 Mar 30. doi: 10.1186/s13071-023-05727-y, PMID: 36998029 PMC10061416

[ref9] Esemu S. N. Ndip L. M. Ndip R. N. (2011). Ehrlichia species, probable emerging human pathogens in sub-Saharan Africa: environmental exacerbation. Rev. Environ. Health 26, 269–279. doi: 10.1515/reveh.2011.034. PMID: 22435325, PMID: 22435325

[ref11] Guo W. P. Tian J. H. Lin X. D. Ni X. B. Chen X. P. Liao Y.. (2016). Extensive genetic diversity of Rickettsiales bacteria in multiple mosquito species. Sci. Rep. 6:38770. doi: 10.1038/srep38770, PMID: 27934910 PMC5146937

[ref13] He L. Bastos R. G. Sun Y. Hua G. Guan G. Zhao J.. (2021). Babesiosis as a potential threat for bovine production in China. Parasit. Vectors 14:460. doi: 10.1186/s13071-021-04948-3, PMID: 34493328 PMC8425137

[ref14] He M. Zhang L. Hu H. Liu X. Zhang C. Xin Y.. (2023). Complete genome sequencing and comparative genomic analyses of a new spotted-fever *Rickettsia heilongjiangensis* strain B8. Emerg. Microbes Infect. 12:2153085. doi: 10.1080/22221751.2022.2153085, PMID: 36440590 PMC9930820

[ref16] Jia N. Zheng Y. C. Jiang J. F. Ma L. Cao W. C. (2013). Human infection with Candidatus Rickettsia tarasevichiae. N. Engl. J. Med. 369, 1178–1180. doi: 10.1056/NEJMc1303004 24047080

[ref17] Jin X. Liao J. Chen Q. Ding J. Chang H. Lyu Y.. (2023). Diversity of Rickettsiales bacteria in five species of ticks collected from Jinzhai County, Anhui Province, China in 2021-2022. Front. Microbiol. 14:1141217. doi: 10.3389/fmicb.2023.1141217, PMID: 37187539 PMC10175684

[ref18] Kang Y. J. Diao X. N. Zhao G. Y. Chen M. H. Xiong Y. Shi M.. (2014). Extensive diversity of Rickettsiales bacteria in two species of ticks from China and the evolution of the Rickettsiales. BMC Evol. Biol. 14:167. doi: 10.1186/s12862-014-0167-2, PMID: 25073875 PMC4236549

[ref20] Kim H. K. (2022). *Rickettsia*-host-tick interactions: knowledge advances and gaps. Infect. Immun. 90:e0062121. doi: 10.1128/iai.00621-21, PMID: 35993770 PMC9476906

[ref21] Kocan K. M. De La Fuente J. Guglielmone A. A. Meléndez R. D. (2003). Antigens and alternatives for control of *Anaplasma marginale* infection in cattle. Clin. Microbiol. Rev. 16, 698–712. doi: 10.1128/cmr.16.4.698-712.2003, PMID: 14557295 PMC207124

[ref23] Li Y. Galon E. M. Guo Q. Rizk M. A. Moumouni P. F. A. Liu M.. (2020). Molecular detection and identification of Babesia spp., Theileria spp., and Anaplasma spp. in sheep from border regions, northwestern China. Front. Vet. Sci. 7:630. doi: 10.3389/fvets.2020.00630, PMID: 33195501 PMC7526627

[ref24] Li H. Zhang P. H. du J. Yang Z. D. Cui N. Xing B.. (2019). *Rickettsia japonica* infections in humans, Xinyang, China, 2014-2017. Emerg. Infect. Dis. 25, 1719–1722. doi: 10.3201/eid2509.171421, PMID: 31441748 PMC6711240

[ref25] Li H. Zhang L. K. Li S. F. Zhang S. F. Wan W. W. Zhang Y. L.. (2019). Calcium channel blockers reduce severe fever with thrombocytopenia syndrome virus (SFTSV) related fatality. Cell Res. 29, 739–753. doi: 10.1038/s41422-019-0214-z, PMID: 31444469 PMC6796935

[ref26] Li H. Zheng Y. C. Ma L. (2015). Human infection with a novel tick-borne Anaplasma species in China: a surveillance study. Lancet Infect. Dis. 15, 663–670. doi: 10.1016/S1473-3099(15)70051-4, PMID: 25833289

[ref27] Li-Juan Z. Zhi-Min X. Si-Min D. Hui D. Shan L. Jing X.. (2018). Endemic status of schistosomiasis in People's Republic of China in 2017. Zhongguo Xue Xi Chong Bing Fang Zhi Za Zhi. 30, 481–488. doi: 10.16250/j.32.1374.2018219 30567015

[ref29] Lu M. Qin X. C. Jiang Y. Z. Guo Q. Jin X. J. Teng Z. Q.. (2023). Emergence of ehrlichiosis by a new tick-borne Ehrlichia species in China. Int. J. Infect. Dis. 131, 32–39. doi: 10.1016/j.ijid.2023.03.038, PMID: 36967037

[ref30] Lu Q. Yu J. Yu L. Zhang Y. Chen Y. Lin M.. (2018). *Rickettsia japonica* infections in humans, Zhejiang Province, China, 2015. Emerg. Infect. Dis. 24, 2077–2079. doi: 10.3201/eid2411.170044, PMID: 30334710 PMC6200003

[ref31] Madison-Antenucci S. Kramer L. D. Gebhardt L. L. Kauffman E. (2020). Emerging tick-borne diseases. Clin. Microbiol. Rev. 33, e00083–e00018. doi: 10.1128/CMR.00083-18 31896541 PMC6941843

[ref32] Mansfield K. L. Jizhou L. Phipps L. P. Johnson N. (2017). Emerging tick-borne viruses in the twenty-first century. Front. Cell. Infect. Microbiol. 7:298. doi: 10.3389/fcimb.2017.00298, PMID: 28744449 PMC5504652

[ref33] Matos A. L. Curto P. Simões I. (2022). Moonlighting in Rickettsiales: expanding virulence landscape. Trop. Med. Infect. Dis. 7:32. doi: 10.3390/tropicalmed7020032, PMID: 35202227 PMC8877226

[ref34] Matsumoto K. Takeuchi T. Yokoyama N. Katagiri Y. Ooshiro M. Zakimi S.. (2011). Detection of the new Ehrlichia species closely related to *Ehrlichia ewingii* from *Haemaphysalis longicornis* in Yonaguni Island, Okinawa, Japan. J. Vet. Med. Sci. 73, 1485–1488. doi: 10.1292/jvms.11-0007, PMID: 21712642

[ref35] Mediannikov O. Fenollar F. (2014). Looking in ticks for human bacterial pathogens. Microb. Pathog. 77, 142–148. doi: 10.1016/j.micpath.2014.09.008, PMID: 25229617

[ref37] Moura de Aguiar D. Pessoa Araújo Junior J. Nakazato L. Bard E. Aguilar-Bultet L. Vorimore F.. (2019). Isolation and characterization of a novel pathogenic strain of Ehrlichia minasensis. Microorganisms 7:528. doi: 10.3390/microorganisms7110528, PMID: 31694172 PMC6921006

[ref39] Niu Q. Liu Z. Yu P. Yang J. Abdallah M. O. Guan G.. (2015). Genetic characterization and molecular survey of Babesia bovis, Babesia bigemina and Babesia ovata in cattle, dairy cattle and yaks in China. Parasit. Vectors 8:518. doi: 10.1186/s13071-015-1110-0 26452623 PMC4600270

[ref40] Rizk M. A. el-Sayed S. A. E. S. Terkawi M. A. Youssef M. A. el Said E. S. E. S. Elsayed G.. (2015). Optimization of a fluorescence-based assay for large-scale drug screening against Babesia and Theileria parasites. PLoS One 10:e0125276. doi: 10.1371/journal.pone.0125276, PMID: 25915529 PMC4411034

[ref42] Teng Z. Gong P. Wang W. Zhao N. Jin X. Sun X.. (2023). Clinical forms of Japanese spotted fever from case-series study, Zigui County, Hubei Province, China, 2021. Emerg. Infect. Dis. 29, 202–206. doi: 10.3201/eid2901.220639, PMID: 36573633 PMC9796216

[ref44] Tian Z. C. Liu G. Y. Shen H. Xie J. R. Luo J. Tian M. Y. (2012). First report on the occurrence of Rickettsia slovaca and *Rickettsia raoultii* in *Dermacentor silvarum* in China. Parasit. Vectors 5:19. doi: 10.1186/1756-3305-5-19, PMID: 22257726 PMC3292836

[ref45] Tokarz R. Lipkin W. I. (2021). Discovery and surveillance of tick-borne pathogens. J. Med. Entomol. 58, 1525–1535. doi: 10.1093/jme/tjaa269, PMID: 33313662 PMC8285023

[ref46] Wang J. Yang J. Gao S. Liu A. Rashid M. Li Y.. (2020). Rapid detection and differentiation of Theileria annulata, T. Orientalis and *T. sinensis* using high-resolution melting analysis. Ticks Tick Borne Dis. 11:101312. doi: 10.1016/j.ttbdis.2019.101312, PMID: 31645296

[ref47] Wen B. Cao W. Pan H. (2003). Ehrlichiae and ehrlichial diseases in China. Ann. N. Y. Acad. Sci. 990, 45–53. doi: 10.1111/j.1749-6632.2003.tb07335.x 12860598

[ref48] Wen B. Jian R. Zhang Y. Chen R. (2002). Simultaneous detection of Anaplasma marginale and a new Ehrlichia species closely related to *Ehrlichia chaffeensis* by sequence analyses of 16S ribosomal DNA in *Boophilus microplus* ticks from Tibet. J. Clin. Microbiol. 40, 3286–3290. doi: 10.1128/JCM.40.9.3286-3290.2002, PMID: 12202567 PMC130830

[ref49] Yousefi A. Rahbari S. Shayan P. Sadeghi-Dehkordi Z. Bahonar A. (2017). Molecular detection of Anaplasma marginale and *Anaplasma ovis* in sheep and goat in west highland pasture of Iran. Asian Pac. J. Trop. Biomed. 7, 455–459. doi: 10.1016/j.apjtb.2017.01.017

[ref50] Zhai J. Wu Y. Chen J. Zou J. Shan F. Li W.. (2021). Identification of Amblyomma javanense and detection of tick-borne Ehrlichia spp. in confiscated Malayan pangolins. Int. J. Parasitol. Parasites Wildl. 14, 107–116. Published 2021 Jan 30. doi: 10.1016/j.ijppaw.2021.01.008, PMID: 33598400 PMC7868807

[ref51] Zhang X. Chen H. Han D. Wu W. (2023). Clinical usefulness of metagenomic next-generation sequencing for Rickettsia and *Coxiella burnetii* diagnosis. Eur. J. Clin. Microbiol. Infect. Dis. 42, 681–689. doi: 10.1007/s10096-023-04586-w, PMID: 36997767 PMC10172222

[ref52] Zhao G. P. Wang Y. X. Fan Z. W. Ji Y. Liu M. J. Zhang W. H.. (2021). Mapping ticks and tick-borne pathogens in China. Nat. Commun. 12:1075:1075. doi: 10.1038/s41467-021-21375-1, PMID: 33597544 PMC7889899

