# Supplementary material for: Molecular detection reveals diverse tick-borne bacterial and protozoan pathogens in two tick species from Yingshan County of Hubei Province, China in 2021–2022
Source: Front Microbiol. 2023 Nov 23;14:1298037. doi: 10.3389/fmicb.2023.1298037 (PMC10701897; doi:10.3389/fmicb.2023.1298037)
Supplement: Supplementary file 1 [file Table_1.DOCX]

Supplementary Material

# Supplementary Figures and Tables

## Supplementary Tables

**Supplementary Table 1.** Primers for the amplification of sequences of ticks and tick-borne pathogens

| Organism | Gene | PCR method | Cycle | Primer name | Sequences | Anticipated amplicon length (bp) | Reference |
| --- | --- | --- | --- | --- | --- | --- | --- |
| Ticks | *CO1* | PCR | 1 | TickCOI-F | GGTCAACAAATCATAAAGATATTGG | 700 | Wang, et al., 2020 |
|  |  |  | 1 | TickCOI-R | TAAACTTCAGGGTGACCAAAAAATCA |  |  |
| *Rickettsia* | 16S rRNA | qPCR |  | R-rrs -F | GGAGCATGCGGTTTAATTCG | _ | Kawamori, et al., 2018 |
|  |  |  |  | R -rrs-R | GCCATGCAACACCTGTGTGT |  |  |
|  |  |  |  | R -rrs-Probe | VIC-CGGATCGCAGAGATG-MGB |  |  |
|  | 16S rRNA | Semi-Nested PCR | 1,2 | R-rrs1-F | GTACGGAATAACTTTTAGAAAT | 900 | Wang, et al., 2020; Guo et al., 2016; Roux et al., 1997 |
|  |  |  | 1 | R-rrs1-R1 | CATGATGACTTGACRTCGT |  |  |
|  |  |  | 2 | R-rrs1-R2 | CATCTCACGACACGAGCTG |  |  |
|  | 16S rRNA | Semi-Nested PCR | 1 | R-rrs2-F1 | GAAGGCGRTCATYTRGGCT | 700 |  |
|  |  |  | 2 | R-rrs2-F2 | GRTCATYTRGGCTRCAACTG |  |  |
|  |  |  | 1,2 | R-rrs2-R | CTGCCTCTTGCGTTAGCT |  |  |
|  | *omp*A | Semi-Nested PCR | 1,2 | R-*omp*A-F | TGGCGAATATTTCTCCAAAA | 500 |  |
|  |  |  | 1 | R-*omp*A-R1 | TGCATTTGTATTACCTATTGT |  |  |
|  |  |  | 2 | R-*omp*A-R2 | AGTGCAGCATTCGCTCCCCCT |  |  |
|  | *gltA* | Semi-Nested PCR | 1 | R-gltA-F1 | CCGGGYTTTATGTCTACTGC | 900 |  |
|  |  |  | 2 | R-gltA-F2 | CTTTATGTCTACTGCKTCTTG |  |  |
|  |  |  | 1,2 | R-gltA-R | AGCTGTCTWGGTCTGCTGATT |  |  |
|  | *groEL* | Semi-Nested PCR | 1 | R-groRL-F1 | CCATTACATGATAGAATTGCAAT | 1100 |  |
|  |  |  | 2 | R-groRL-F2 | GAATTGCAATAAAGCCTATCG |  |  |
|  |  |  | 1, 2 | R-groRL-R | CCATCATTGCTTTTCTTCTATC |  |  |
| Anaplasmataceae | 23S rRNA | qPCR |  | Ana-F | TGA CAG CGT ACC TTT TGC AT | _ | Ngnindji et al., 2022 |
|  |  |  |  | Ana-R | GTA ACA GGT TCG GTC CTC CA |  |  |
|  |  |  |  | Ana-Probe | GGA TTA GAC CCG AAA CCA AG |  |  |
|  | 16S rRNA | Nested PCR | 1 | A&E-rrs-F1 | GAA CGA ACG CTG GCG GCA AGC | 500 | Jafar et al., 2018 |
|  |  |  | 1 | A&E-rrs-R1 | AGT A(T/C)C G(A/G)A CCA GAT AGC CGC |  |  |
|  |  |  | 2 | A&E-rrs-F2 | TGC ATA GGA ATC TAC CTA GTA G |  |  |
|  |  |  | 2 | A&E-rrs-R2 | CTA GGA ATT CCG CTA TCC TCT |  |  |
| *Anaplasma spp.* | 16S rRNA | Semi-Nested PCR | 1,2 | ANArrs1-F | GGATAGCCACTRGAARTGGTG | 800 | Guo et al., 2018 |
|  |  |  | 1 | ANArrs1-R1 | CGTGCTGACTTGACATCAT |  |  |
|  |  |  | 2 | ANArrs1-R2 | CATCTCACGACACGAGCTG |  |  |
|  |  | Semi-Nested PCR | 1 | ANArrs2-F1 | CTGTCTGGTCCGGTACTGAC | 800 |  |
|  |  |  | 1，2 | ANArrs2-R | TGCCTCCTTDCGGTTGGC |  |  |
|  |  |  | 2 | ANArrs2-F2 | TGGTCCGGTACTGACRCT |  |  |
| *A. marginale* | *gltA* | Semi-Nested PCR | 1 | A.m-gltA-F1 | TGGTAGAAAAAGCGATTTTAG | 1200 | Lu et al., 2022 |
|  |  |  | 1,2 | A.m-gltA-R | CCGGTATAAAGTTGGCGT |  |  |
|  |  |  | 2 | A.m-gltA-F2 | ATAAGCTTGCCCGTTATGC |  |  |
|  | *groEL* | Semi-Nested PCR | 1 | A.m-groEL-F1 | ACATGCTCCATACTGACTGC | 850 |  |
|  |  |  | 1,2 | A.m-groEL-R | AGATGCAAGCGTGTATAGCAG |  |  |
|  |  |  | 2 | A.m-groEL-F2 | AGATGAGATTGCACAGGTTG |  |  |
| *A. platys* | *gltA* | Semi-Nested PCR | 1 | A.p-gltA-F1 | TGRAAGAAAAWGCTGTTTTG | 850 | Guo et al., 2016 |
|  |  |  | 1,2 | A.p-gltA-R | GCTCTRGGRTCATARCTYTT |  |  |
|  |  |  | 2 | A.p-gltA-F2 | AGCTRTTTTRGAGTGYGGAG |  |  |
|  | *groEL* | Semi-Nested PCR | 1 | A.P-groEL-F1 | AGTCGATTAGGGAAGTAGTAC | 800 |  |
|  |  |  | 1,2 | A.P-groEL-R | GCGTCCTCTACTCTGTCTT |  |  |
|  |  |  | 1 | A.P-groEL-F2 | AGGATGGCTACAAGGTAATG |  |  |
| *A. bovis* | *gltA* | Semi-Nested PCR | 1 | A.b-gltA-F1 | TTYATAGATGGRGATRAGGGC | 400 | Guo et al., 2019 |
|  |  |  | 1,2 | A.b-gltA-R1 | AHCATTTCATRCCAYTGRG |  |  |
|  |  |  | 2 | A.b-gltA-F2 | AGATGGRGATRAGGGCATYCT |  |  |
|  | *groEL* | semi-nested pcr | 1,2 | A.b-groEL-F | TTGCTAAATCTGGAAGRCCAC | 800 |  |
|  |  |  | 1 | A.b-groEL-R1 | GARGACGTTGAGGGTGAAGC |  |  |
|  |  |  | 2 | A.b-groEL-R2 | CATAAATACYGCCGCRAGAG |  |  |
| *Ca. A. boleense* | *gltA* | Semi-Nested PCR | 1 | Ca.b-gltA-F1 | GYAGCATAGCGYATTTGTTGTTG | 700 | Lu et al., 2022 |
|  |  |  | 1,2 | Ca.b-gltA-R | TCAACRTTAGGGTAAAGCTTGCG |  |  |
|  |  |  | 2 | Ca.b-gltA-F2 | GYAGCATAGCGYATTTGTTGTTG |  |  |
|  | *groEL* | Semi-Nested PCR | 1 | Ca.b-groEL-F1 | CCGGAAATCACAAAAGACG | 800 |  |
|  |  |  | 1,2 | Ca.b-groEL-R | AATACTTTCGGAATTACTATCTAC |  |  |
|  |  |  | 2 | Ca.b-groEL-F2 | TATGRTCRAAGAAGCAGTATT |  |  |
| *Ehrlichia* spp. | 16S rRNA | Semi-Nested PCR | 1,2 | Ehrrs1-F | GAATAGCCATTAGAAATGATG | 700 | Guo et al., 2018 |
|  |  |  | 1 | Ehrrs1-R1 | GTCAGTATCGAACCAGATAG |  |  |
|  |  |  | 2 | Ehrrs1-R2 | GTATCGAACCAGATAGCCG |  |  |
|  | 16S rRNA | Semi-Nested PCR | 1 | Ehrrs2-F1 | CGGCTATCTGGTTCGATAC | 800 |  |
|  |  |  | 1,2 | Ehrrs2-R | GCTTCCTTKCGGTTAGCAC |  |  |
|  |  |  | 2 | Ehrrs2-F2 | CTATCTGGTTCGATACTGAC |  |  |
|  | *glt*A | Semi-Nested PCR | 1 | EhgltA-F1 | CAGGHTTTATGTCWACTGCTGCT | 1000 | Teng et al., 2023 |
|  |  |  | 1,2 | EhgltA-R | TAYAAYTGACGWGGACGACAT |  |  |
|  |  |  | 2 | EhgltA-F2 | TTATGTCWACTGCTGCTTGTGA |  |  |
|  | *groEL* | Nested PCR | 1 | EhgroEL-F1 | CGYCAGTGGGCTGGTAATGAA | 800 | Michelet et al., 2014 |
|  |  |  | 1 | EhgroEL-R1 | CCWCCWGGTACWACACCTTC |  |  |
|  |  |  | 2 | EhgroEL-F2 | TGGCAAATGTAGTTGTAACAGG |  |  |
|  |  |  | 2 | EhgroEL-R2 | GCCGACTTTTAGTACAGCAA |  |  |
| *Babesia–Theileria* | 18S rRNA | nested PCR | 1 | BTH18S-F1 | GTGAAACTGCGAATGGCTCATTAC | 1400 | Kumar t et al., 2022 |
|  |  |  | 1 | BTH18S-R1 | AAGTGATAAGGTTCACAAAACTTCCC |  |  |
|  |  |  | 2 | BTH18S- F2 | GGCTCATTACAACAGTTATAGTTTATTTG |  |  |
|  |  |  | 2 | BTH18S- R2 | CGGTCCGAATAATTCACCGGAT |  |  |

# Reference

Wang, J., Yang, J., Gao, S., Liu, A., Rashid, M., Li, Y., et al. (2020). Rapid detection and differentiation of Theileria annulata, T. Orientalis and T. sinensis using high-resolution melting analysis. Ticks Tick Borne Dis. 11:101312. doi: 10.1016/j.ttbdis.2019.101312

Kawamori, F., Shimazu, Y., Sato, H., Monma, N., Ikegaya, A., Yamamoto, S., et al. (2018). Evaluation of diagnostic assay for rickettsioses using duplex real-time PCR in multiple Laboratories in Japan. Jpn. J. Infect. Dis. 71, 267–273. doi: 10.7883/yoken. JJID.2017.447

Guo, W. P., Tian, J. H., Lin, X. D., Ni, X. B., Chen, X. P., Liao, Y., et al. (2016). Extensive genetic diversity of Rickettsiales bacteria in multiple mosquito species. Sci. Rep. 6:38770. doi: 10.1038/srep38770

Roux, V., Rydkina, E., Eremeeva, M., and Raoult, D. (1997). Citrate synthase gene comparison, a new tool for phylogenetic analysis, and its application for the rickettsiae. Int. J. Syst. Bacteriol. 47, 252–261. doi: 10.1099/00207713-47-2-252

Ngnindji-Youdje, Y., Diarra, A. Z., Lontsi-Demano, M., Tchuinkam, T., and Parola, P. (2022). Detection of tick-borne pathogens in ticks from cattle in Western highlands of Cameroon. Microorganisms 10:1957. doi: 10.3390/microorganisms10101957

Jafar, B. A., Ramzgouyan, M. R., Shirian, S., Faghihi, F., Bakhshi, H., Naseri, F., et al. (2018). Molecular characterization and phylogenetic analysis of Anaplasma spp. and Ehrlichia spp. isolated from various ticks in southeastern and northwestern regions of Iran. Vector Borne Zoonotic Dis. 18, 252–257. doi: 10.1089/vbz.2017.2219

Guo, W. P., Huang, B., Zhao, Q., Xu, G., Liu, B., Wang, Y. H., et al. (2018). Human pathogenic Anaplasma spp., and Rickettsia spp. in animals in Xian, China. PLoS Negl. Trop. Dis. 12:e0006916. doi: 10.1371/journal.pntd.0006916

Lu, M., Meng, C., Gao, X., Sun, Y., Zhang, J., Tang, G., et al. (2022). Diversity of Rickettsiales in Rhipicephalus microplus ticks collected in domestic ruminants in Guizhou Province, China. Pathogens 11:1108. doi: 10.3390/pathogens11101108. PMID: 36297165; PMCID: PMC9607482

Guo, W. P., Wang, X., Li, Y. N., Xu, G., Wang, Y. H., and Zhou, E. M. (2019). GroEL gene typing and genetic diversity of Anaplasma bovis in ticks in Shaanxi. China. Genet. Infect. Dis. 74:103927. doi: 10.1016/j.meegid.2019.103927

Teng, Z., Shi, Y., Zhao, N., Zhang, X., Jin, X., He, J., et al. (2023b). Molecular detection of tick-borne bacterial and protozoan pathogens in Haemaphysalis longicornis (Acari: Ixodidae) ticks from free-ranging domestic sheep in Hebei Province, China. Pathogens 12:763. doi: 10.3390/pathogens12060763

Michelet, L., Delannoy, S., Devillers, E., Umhang, G., Aspan, A., Juremalm, M., et al. (2014). High-throughput screening of tick-borne pathogens in Europe. Front. Cell. Infect. Microbiol. 4:103. doi:10.3389/fcimb.2014.00103

Kumar, B., Maharana, B. R., Thakre, B., Brahmbhatt, N. N., and Joseph, J. P. (2022). 18S rRNA gene-based Piroplasmid PCR: an assay for rapid and precise molecular screening of Theileria and Babesia species in animals. Acta Parasitol. 67, 1697–1707. doi: 10.1007/s11686-022-00625-2
